# Impact of Digital Device, Exercise, and Music Intervention Programs on the Cognition and Depression of the Elderly in South Korea: A Meta-Regression Analysis

**DOI:** 10.3390/ijerph19074036

**Published:** 2022-03-29

**Authors:** Jaeeon Yoo, Junga Oh, Sang-Youn Kim, Jungmin Shin, Siekyeong Kim, Changhyun Roh

**Affiliations:** 1 Department of Social Welfare, Gachon University, 1342 Seongnamdaero, Sujeong-gu, Seongnam-si 13120, Korea; jejowa0205@gachon.ac.kr; 2 Chungcheongnam-do Public Agency for Social Service, 10-22, Yehak-ro, Sapgyo-eup, Yesan-gun 32416, Korea; 3 Interaction Laboratory, Advanced Technology Research Center, Korea University of Technology and Education, Cheonan-si 31253, Korea; sykim@koreatech.ac.kr (S.-Y.K.); jmshin@koreatech.ac.kr (J.S.); 4 Department of Psychiatry, Chungbuk National University, College of Medicine and CBNU Hospital, 1 Chungdae-ro, Cheongju-si 28644, Korea; poshong@chungbuk.ac.kr; 5 Korea Atomic Energy Research Institute, 111, Daedeok-daero 989beon-gil, Yuseong-gu, Daejeon 34057, Korea; chroh@kaeri.re.kr

**Keywords:** cognition, dementia, depression, program

## Abstract

Background: This study examined the effect of digital devices, exercise, and music intervention programs for the elderly in Korea on their cognition and depression. Methods: This study selected 70 cognition programs and 46 depression programs for the elderly in Korea. This study controlled the characteristics of the programs and participants, and conducted a meta-regression analysis to estimate the intervention effect size of digital devices, exercise, and music on cognition and depression. Results: The meta-regression analysis revealed that digital device programs had a smaller effect size with respect to the improvement of cognitive functions than programs that did not use digital devices. The exercise programs had a small effect size on depression, but their effect size on cognition was not significantly different. Discussion: These findings provide implications for developing a program that combines music therapy with digital devices and exercise interventions, which can be effective in addressing both cognition and depression.

## 1. Introduction

As the size of the South Korean elderly population is increasing at the fastest rate in the world, the government and populace of the nation are devoting more attention to the prevention and management of dementia. Statistics Korea [1] announced that the South Korean elderly population has reached almost 10 million and it will account for 20% of the total population by 2026, which would make the nation a super-aged society. It is also forecasted that the elderly population will continue to increase to 19 million, which would be 40% of the total population, by 2051 [1]. The National Institute of Dementia of the National Medical Center also published an epidemiological report that projected that the number of dementia patients with senile diseases will reach 3.07 million and social costs will be KRW 107 trillion (approximately USD 100 billion) by 2050 [2]. The South Korean government recognizes this situation as a serious social problem and has made active efforts to facilitate the prevention, diagnosis, management, and treatment of dementia. Additionally, it has also enabled dementia-related education, research and development, and publicity. For instance, it announced the National Responsibility Policy for Dementia Care in 2017 and the Forth Dementia Management Plan in 2021.

It is necessary to develop intervention approaches that effectively address cognition and depression in the elderly in order to reduce their burden on caregivers and social costs, in addition to improving the health and well-being of the elderly. Dementia is a degenerative disease, and it is particularly painful for patients and their families [3]. Dementia, which mainly afflicts the elderly, causes cognitive dysfunction and deteriorates a person’s ability to live independently [4]. The pain caused by dementia often leads to depression, which further facilitates cognitive decline [5]. To make it worse, the South Korean state and society are expected to face an increased burden with regard to dementia management, as the rapid decrease of its young population signifies that there will be fewer available formal and informal caregivers to care for the elderly with dementia in the future [6].

Non-pharmacological intervention programs that address cognition and depression have been attracting the attention of clinical practice and several researchers [7]. Non-pharmacological interventions refer to the psychological and social approaches used to alleviate the symptoms of a disease and improve quality of life that do not involve the use of drugs [8]. They apply a variety of single or integrated methods, including digital devices, sports, music, art, gardening, work, and retrospection [9]. Social and economic burdens have been increasing due to the increase in the number of dementia patients. However, there is still no known treatment that can restore a dementia-afflicted individual’s decline in cognitive function to a normal level [7,8]. Even though it is difficult, if not impossible, to cure dementia, it is possible to delay its progress through non-pharmacological interventions in its early stages [9]. Consequently, the need for non-pharmacological interventions to prevent, alleviate, and delay dementia and improve mental health has increased. Non-pharmacological interventions are effective in maintaining cognitive functions and stabilizing emotions by inducing patients to perform tasks in a familiar environment [10]. In addition to being used for the treatment of dementia itself, the demand for non-pharmacological interventions that could improve the leisure and quality of life of the elderly with dementia is also increasing [8]. Such programs have been developed and services have been provided in accordance with academic expertise in various specialized fields, which include health, medical care, nursing, social welfare, exercise rehabilitation, and music [7,9]. Moreover, many empirical studies have demonstrated their effectiveness [10].

In particular, (digital) equipment, exercise, and music programs have spread widely, but their effectiveness in comparison to other intervention programs remains uncertain. For example, Dementia Support Centers, which have been established in all 256 municipal administrative districts in South Korea, have attempted to utilize digital devices, such as artificial intelligence, virtual reality, information and communication technology, and robots, in cognitive stimulation and rehabilitation programs [11]. These Dementia Support Centers are stipulated to operate in accordance with the guidelines of the Ministry of Health and Welfare and are obligated to provide exercise and music programs [11].

The development and implementation of intervention programs that incorporate these devices and technologies have been relatively uncommon, because the elderly in South Korea were unfamiliar with digital devices until a few years ago [12]. Although there are only a handful of studies on the utilization of digital devices, recent studies have developed digital devices, utilized them practically, and evaluated their effectiveness [13]. Several previous studies have reported that digital devices can maintain and improve cognitive function in the elderly with dementia or reduce depression [14]. A few recent studies have developed programs by grafting older programs, and found that they were effective. We have also been developing similar programs, so it is necessary to compare the effect sizes of existing programs.

Several studies on exercise and music therapy, which are non-pharmacological interventions, have shown that they have a positive impact on behavioral problems, anxiety behavior, emotional state, anxiety and depression, cognitive ability, communication ability, socialization ability, and the quality of life of the elderly with dementia [15,16,17]. Exercise programs, which have been developed primarily to facilitate the maintenance of people’s physical and cognitive functions, make participants perform physical movements repetitively [15]. However, most exercise programs have ignored the elements that interested and pleased the participants [17]. Unlike exercise programs, music programs in which participants solely listened to music did not facilitate the maintenance of their physical function [18]. Playing real musical instruments, singing, and/or dancing were also attempted, but these activities presented multiple challenges [18]. Playing a real musical instrument was difficult to implement as a group program. Additionally, there were difficulties related to playing the instrument, the instrument’s cost, and its weight. The elderly with sound cognitive function or those with mild dementia can participate in music programs that involve dancing, but the elderly with severe dementia and elderly patients at facilities/hospitals face a serious risk of injuring themselves when participating in such programs [19].

The objectives of this study were to accumulate and analyze the achievements of researchers who have sought to design and/or evaluate more effective cognition and depression programs and identify innovative intervention methods. Since programs that address the cognition and depression of the elderly have been actively developed and evaluated in South Korea in recent years, many studies have evaluated the effectiveness of digital device programs, exercise programs, and music programs [14,17,18,19]. However, it is difficult to determine the effectiveness of the three aforementioned varieties of programs in comparison to other programs [18]. Although programs that incorporate specific activities, such as the use of digital devices, exercise, or music, have been developed and implemented, it is necessary to shift the approach to consider all of these elements comprehensively [10,18]. The number of formal and informal care providers will inevitably decrease and social costs will increase in South Korea over the next few decades, and the government and service-providing agencies have been attempting to increase the utilization of digital devices [6]. To explore ways of improving their effectiveness, this study evaluates the impact of digital device programs, exercise programs, and music programs on the cognition and depression of the elderly. We have been developing a digital device that integrates virtual reality and haptic sticks. Moreover, we have been working on a project to devise and apply an exercise and music program that utilizes this device. As a preliminary step for planning the curriculum of an exercise and music program, this study conducted a meta-regression analysis to comprehensively grasp the effectiveness of all previously implemented and verified programs. More specifically, this study compared the effect sizes of digital device programs, exercise programs, and music programs while controlling for the characteristics of the South Korean agencies and elderly participants who organized or participated in the 70 cognition-related and 46 depression-related programs, which compared the effectiveness of these programs by comparing the results before and after their implementation and the results of the control group. The specific research questions were as follows:

*Research Question 1: What are the heterogeneity and risk of bias of intervention programs targeting the elderly in South Korea on cognition and depression?*
*Research Question 2: How do the effect sizes of programs targeting the elderly in South Korea vary when digital devices, exercise, and music are incorporated?*

## 2. Method

### 2.1. Eligibility Criteria

The subjects of this study’s meta-regression analysis were non-pharmacological intervention programs for the elderly that were conducted from 2008 to 2020 to improve their cognitive function and alleviate depression. The long-term care insurance system for the elderly has been in effect in South Korea since 2008. Moreover, various service providers and programs have been operated and managed in accordance with the Dementia Management Act [11]. Before the implementation of the long-term care insurance system for the elderly and the Dementia Management Act, it was difficult to guarantee the quality of reports on the effect size of intervention programs for the elderly, because their curricula and effectiveness were not systematically managed by the public. Therefore, programs prior to 2008 were excluded. Programs that utilize digital devices have been implemented and studied quite recently [12,13,15]. Consequently, this study included studies published in 2020 in order to analyze as many publications as possible. This study limited the spatial scope to South Korea—excluding other countries and Korean–American subjects—but it did not have any restrictions with respect to where these studies were published [10,17,19]. Since this study targeted the elderly who suffer from cognitive decline or dementia, it excluded studies that also examined subjects with normal cognitive functions and those that evaluated adults who were 50 years old or younger [10,15,18]. Studies whose subjects were people over the age of 50 were selected. Programs in which middle-aged and elderly people with dementia or at least in cognitive decline were the subject of analysis in this study. This study also did not analyze programs that involved a medical intervention curriculum, including drug therapy [7,8]. However, this study analyzed all non-pharmacological intervention programs, particularly those that utilized digital devices, exercise, music, or a combination of these elements, which were used as independent variables. Although the scope of the curriculum was wide, this study only included studies that conducted evaluations before and after the target programs’ implementation using a measurement tool with proven reliability and validity to ensure the quality of the target programs [15,17,18]. Effective programs with only an experimental group and no control group were excluded from this study’s analysis. In all of the programs we analyzed, the experimental group was randomly assigned so that there was no statistical difference in sociodemographic characteristics from the control group. For studies in which depression or cognitive levels were measured multiple times after the program, the values measured at the point closest to the final session were used.

### 2.2. Data Collection

**Figure 1:**
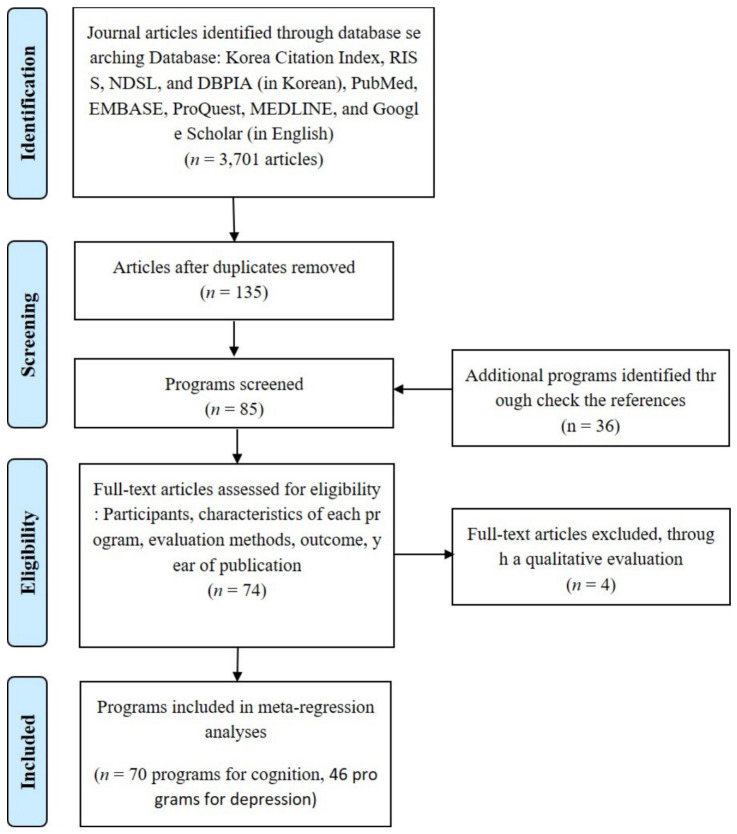
Flow diagram—data collection and selection process.

This study involved a rigorous process of searching, collecting, selecting, coding, standardizing the effect size, and statistically analyzing publications by referring to the Preferred Reporting Items for Systematic Review and Meta-Analysis (PRISMA) [19,20] (Figure 1). Data collection for the meta-analysis was conducted through an online search. The Korea Citation Index, RISS, NDSL, and DBPIA were searched to retrieve relevant articles published in South Korea [14,17,21]. All publications written in Korean or English were published in a Korean academic journal after successfully going through an anonymous peer-review process. Since all publications were published on KCI sites, the selective bias problem did not exist. However, we were concerned that some publications might be omitted due to the algorithm of the search term when using only the KCI. Therefore, we also used RISS, NDSL, and DBPIA, which searched publications using other algorithms; additionally, for cross-validation, even if they did not publish all journal papers. Google, PubMed, EMBASE, ProQuest, and MEDLINE were searched to retrieve relevant articles published in other countries [15,18,22]. These academic publication databases were used to include papers written in English and published in international journals while targeting Koreans. This study included only peer-reviewed journal articles and excluded dissertations, conference presentations, and reports from public institutions. The keywords used for the literature search were: dementia, cognition, depression, elderly, program, treatment, service, digital, exercise, music, dementia program, cognitive program, dementia service, cognitive service, and effect.

Duplicate articles were removed from those that were retrieved from the databases. We then read the titles and abstracts, and excluded publications that were clearly determined to be irrelevant to the analysis that this study aimed to conduct [20]. We also examined the references of the studies that were selected to identify additional relevant articles. Additionally, we repeated the process of searching for new high-priority papers by using the citation and relevance functions of Google Scholar on the retrieved papers until no new papers were found [15,21,23].

The researchers of the present study recorded the bibliographic information of all searched articles and the reasons for the exclusion of each study. When it was not clear whether a program met the selection criteria, the selection of the program was determined through a cross-validation process or discussion among the researchers [10].

The initial search identified 3701 papers, and 135 papers were selected based on their study objectives, subjects, and dependent variables. These papers were excluded because their relevance to this study was notably low, they used drugs, young people under 40 years participated in these studies, and they evaluated effectiveness using dependent variables other than cognition and depression. Among the 135 papers, this study excluded another 50 papers that had only experimental groups without a control group, evaluated only once after implementation without measuring dependent variables before the execution of the program, and examined cognition and depression subjectively without using an objective index. Subsequently, 85 papers were selected because they conducted randomized pre- and post-test comparisons, but 11 of them were excluded because they had insufficient data to calculate the effect size of the program they examined. The papers excluded in this stage did not present the means and standard deviations of the dependent variables before and after implementation, did not provide the location and frequency of a program, or did not show detailed figures for the gender and age of the participants. This study also qualitatively analyzed the selected papers to increase the validity of this study’s results [15,20]. This was conducted to determine whether the primarily selected studies were conducted using a research method that was appropriate for its objective, and it increased the validity of this study [23,24]. The qualitative evaluation of the target papers was conducted based on a non-randomized experimental design evaluation list [20,23,24]. The detailed items of the evaluation were: the appropriateness of the research topic and concept definition, random assignment, enforcement of the blind procedure, blind measures, treatment of the experimental and control groups, variable measurement, statistical analysis methods and reports, total analysis of initial subjects, dropout rate, and place of implementation [15,22]. Four papers were excluded based on this qualitative evaluation. As a result, this study selected 70 cognition-related and 46 depression-related programs as the ultimate targets of its analysis. Among the finally selected papers, some papers measured both cognition and depression, while the other papers examined cognition or depression. Therefore, the number of cognition programs and that of depression programs were different.

### 2.3. Data Items

#### 2.3.1. Dependent Variables: Cognition and Depression

This study also used cognition and depression levels, which were mainly measured to evaluate the effectiveness of cognitive programs that targeted the elderly, as dependent variables. Since the previous studies used different measurement tools, this study converted them into Hedge’s g—a measure of effect size—to standardize and compare effect sizes. The Hedge’s g was calculated using the mean, standard deviation, and sample size of the results related to the cognition or depression of the treatment and control groups before and after the test [25]. It was determined that Hedge’s g was a more appropriate measure than Cohen’s d because the latter tended to overestimate the effect size for studies with a small sample size, which was common among experimental studies [26]. The Hedge’s g value represents the standardized effect size. In this study, a higher cognitive score indicated a higher cognitive level. Therefore, the depression score was converted accordingly, so a higher depression score indicated a lower depression level.

#### 2.3.2. Independent Variables: Digital Devices, Exercise, and Music Programs

We used digital devices, the curriculum of exercise programs, and music programs as major interventional independent variables to explain the heterogeneity of the effect sizes of non-pharmacological intervention programs for the elderly on cognition and depression. For digital devices, it was coded as 1 if a study developed a digital device or software, or used them as the main tool of an intervention program; otherwise, it was coded as 0. For exercise programs, it was coded as 1 if the curriculum of an exercise program conducted at least one exercise session as a main activity and exercise was specified in the program name, paper title, abstract, keywords, or objective; otherwise, it was coded as 0. For music programs, it was coded as 1 if a music program conducted at least one session that involved listening to music, singing, dancing, or playing a musical instrument as its main activity; otherwise, it was coded as 0. Some programs were not coded as ‘yes’ in only one of digital device, exercise, and music, but were coded as ‘yes’ in two or more. Other programs were all coded as ‘no’ in the digital device, exercise, and music variables. The sum of ‘yes’ in the variables of digital device, exercise, and music was not equal to the total sample sizes.

#### 2.3.3. Control Variables: The Characteristics of the Programs and Participants

Since many previous studies have reported that program characteristics (e.g., location and the number of sessions) and participant characteristics (e.g., gender and age) affected the effect size of the program on the participants’ cognition and depression, this study used these characteristics as control variables in addition to the other independent variables (i.e., digital devices, exercise, and music) [10,15,17,24]. The location of the program was coded as 1 if it was a medical facility (e.g., nursing hospital, general hospital, or health center) and 0 if it was another place (e.g., home, nursing home, or senior welfare center). Medical facilities that are operated by professional medical staff receive public health insurance benefits in South Korea, whereas other facilities have different systems, workforce, and support systems, such as the Long-Term Care Insurance for the Elderly. A program’s location, service, and curriculum, as well as the varying characteristics of providers and users, can affect its effect size. The results of the meta-analysis of [10] showed that a program’s location in a medical facility or otherwise also affected its effect size. The number of sessions, coded as a continuous variable, indicates the total number of times intervention was provided to the participants. Gender was coded as 1 when 50% or more of the participants were women and 0 when less than 50% of them were women. Age was a criterion for classifying the older-old group. It was coded as 1 when the participants were 75 years or older, which is when the occurrence of dementia abruptly increases, and 0 when the participants were younger than 75 years.

### 2.4. Statistical Analysis

**Figure 2:**
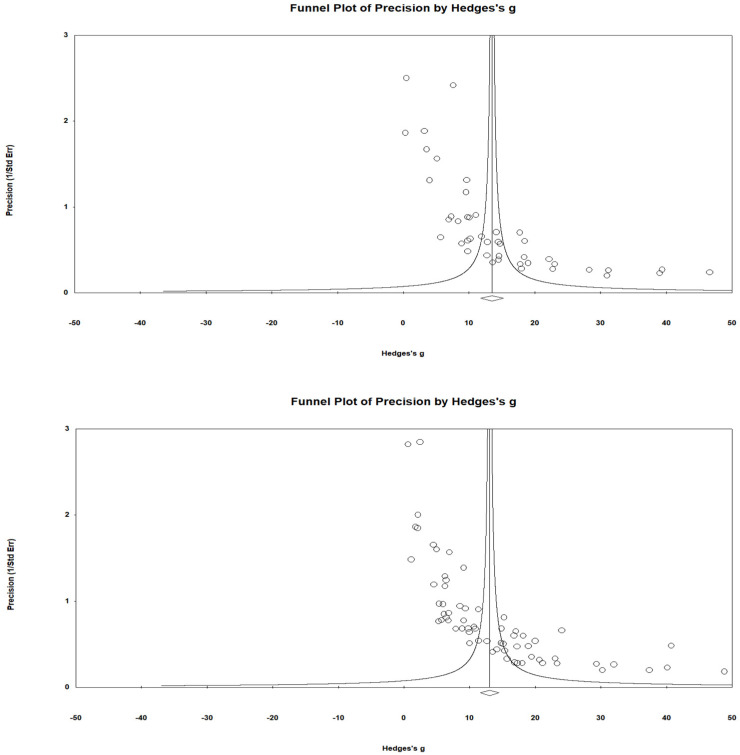
Funnel plot for testing publication bias and the validity of the results: depression (**Top**) and cognition (**Bottom**).

This study was coded using Microsoft Excel to extract the characteristics of the target programs and participants and calculate their effect size for the data analysis. Descriptive statistics were utilized for the characteristics of the target programs and participants (Table 1). An analysis of the risk of publication bias is presented following the results in the general meta-analysis studies based on the PRISMA methodology. In this study, after examining whether the problem of selection bias (Table 2) and bias occurred with a funnel plot, meta-regression analysis was performed to reveal the related factors; the results are shown in Figure 2 and Table 3.

Comprehensive Meta-Analysis 3.3 (Biostar, Englewood, NJ, USA) was used to evaluate the combined effect size of the target studies, the heterogeneity of the studies, the possibility of publication bias of the meta-analysis results, and to perform the meta-regression analysis. Cochran’s *Q* value and Higgins and Green’s *I*^2^ [20] were calculated for the overall effect size and heterogeneity analysis. Cochran’s *Q* value (combined estimate) has a null hypothesis that the effect size of all individual studies is the same [27]. If Cochran’s *Q* value is larger than the df value and the null hypothesis is rejected, the overall effect size can be determined as heterogeneous [27]. *I*^2^ is the ratio of the actual variance to the variance of the total effect size [28]. If it is less than 25%, the heterogeneity is interpreted to be small. If it is 25% or higher and less than 75%, the heterogeneity is considered medium. If it is 75% or more, the heterogeneity is interpreted to be very large. Given that there is a limit to the determination of the heterogeneity based solely on statistical values, researchers need to determine heterogeneity by considering the subject of the study, the type of intervention, and the measurement method, and select a fixed-effects model or a random-effects model [27,28,29]. This study chose a random-effects model, because the target studies used different samples and intervention methods, and each study assumed unequal population effects in independent environments [29].

The symmetry of the funnel plot was also used to determine the publication bias of the meta-analysis results. After visually checking the symmetry using a funnel plot, the possibility of errors was examined using Egger’s regression analysis [30] (Figure 2). This test was also performed because it could indicate the significance of the bias more accurately. In this test, if the relationship between the effect size and standard error is significant, the null hypothesis is rejected. The possibility of publication bias affecting the results was also more thoroughly examined using the trim-and-fill method [31]. It shows the effects of missing data as a difference in the effect size when the asymmetric funnel plot is converted to a symmetrical form [31].

This study conducted a random-effect meta-regression analysis that included moderating variables and control variables to determine the characteristics of the programs for the elderly with dementia that explained the heterogeneity of the effect size [30] (Table 3). Meta-regression analysis is suitable for identifying factors influencing effect sizes when the effect sizes may have heterogeneity [29]. Although sub-group analysis is sometimes used as a method to understand the heterogeneity of effect sizes, it has a limitation in that it is difficult to simultaneously input many explanatory variables into one model [25]. In contrast, meta-regression analysis has the advantage of being able to estimate the association of each explanatory variable with the dependent variable by inputting the characteristics of the program and participants as explanatory variables in one model [29]. Some programs among the analysis targets of this study coded two or more independent variables as 1. Therefore, meta-regression analysis, rather than sub-group analysis, can estimate accurate estimates while considering the association with other independent variables. In the meta-regression analysis, cognition and depression were used as dependent variables for each model. Digital devices, exercise programs, and music programs were used equally as independent variables, and the characteristics that had moderating effects on the effect size were those that affected cognition and depression. Program characteristics (i.e., location and the number of sessions) and participant characteristics (i.e., gender and age)—the variables that were reported to have moderating effects on cognition and depression in previous studies—were used as control variables in the meta-regression analysis. Random-effect meta-regression analysis can be viewed as a type of multiple regression analysis. Therefore, this study presented the characteristics of the entire analysis target (Table 1) and then the analysis results of the final meta-regression analysis (Table 3) in a way similar to multiple regression analysis.

## 3. Results

### 3.1. Study Characteristics

Table 1 shows the characteristics of the intervention programs conducted in the target studies. There were two dependent variables (cognition and depression) and the numbers of programs, analysis targets, were 70 and 46 for cognition and depression, respectively. Therefore, Table 1 shows the overall mean and ratios, and not the characteristics of each individual program.

There were 46 and 70 programs that measured depression and cognition, respectively, as dependent variables. Among the programs that measured depression, 15.2%, 34.8%, and 32.6% of the programs utilized digital devices, exercise, and music, respectively. Programs that measured depression as a dependent variable were mostly exercise programs, but music programs were a close second. However, there were fewer depression-related programs that used digital devices. Half (50.0%) of the programs were conducted in medical facilities, and the mean number of sessions was 16.3. Moreover, 78.3% of the programs had 50% or more female participants, and the mean age of participants was 75 years or older in 67.4% of the programs.

Among programs that measured cognition as a dependent variable, 11.4%, 46.6%, and 32.8% of them utilized digital devices, exercise, and music, respectively. When compared to the programs that measured their effect size on depression, exercise programs were more abundant, whereas digital devices were infrequently utilized. Although it is hard to argue conclusively, it can be said that the significant effect size of digital devices on cognition has not been researched adequately, especially compared to the large number of studies that use exercise to measure cognition as a dependent variable. Among the characteristics of the programs used as control variables, 52.9% of the programs were conducted in medical facilities, and the mean number of sessions was 20.8. Moreover, 82.9% of the programs had 50% or more female participants, and the mean age of the participants was 75 years or older in 61.4% of the programs.

### 3.2. Results of Syntheses

Although the funnel plot that indicates the effect size of individual studies and the overall effect size is in alignment with the overall direction, it is necessary to examine the heterogeneity of the effect size quantitatively, because the degree of heterogeneity between individual studies is only visually evaluated. This study examined Cochran’s *Q*, the *I^2^* value, and *tau*^2^—the variance between studies—to evaluate heterogeneity accurately [27,28]. When the effect size heterogeneity was analyzed statically in the model using depression as a dependent variable, the *Q*-value was 1005.77 (*df* = 38, *p* < 0.001) and the *I*^2^ value was 96.22% (standard: ≥75%), indicating that the heterogeneity was large. The variance between studies (*Tau^2^*) was also high (44.11). In the model using cognition as a dependent variable, the *Q*-value was 1861.44 (*df* = 62, *p* < 0.01) and the *I*^2^ value was 96.67% (standard: ≥75%), indicating that the heterogeneity was large. *Tau*^2^ was also high (39.66). This study chose a random-effects model owing to the diversity of the examined intervention methods and the heterogeneity of their measurement methods.

### 3.3. Risk of Bias across Studies

A publication bias analysis was performed to verify the validity of the results. First, it was assessed using funnel plot analysis, which is a tool used to visually detect the possibility of errors [25,29]. The presence of bias was confirmed because the funnel plot distribution of the random effect model was asymmetric. The probability of the Egger’s regression test regarding depression and cognition was 0.000, and the null hypothesis was rejected. The results showed that the effect sizes and standard errors were significantly different, and the effect size varied based on the sample size. It was judged that there was a publication error in the total effect size; therefore, a trim-and-fill analysis was also conducted [31]. It was assumed that the effect sizes were not reported due to publication error, and this study estimated the random effect size of the modified model when they were added. However, the results of the analysis revealed that the effect sizes were significant, even if they decreased to 6.04 for depression and 5.31 for cognition. In other words, the effect size derived by the meta-regression analysis model of this study was significant, even if there was a possibility of publication error.

### 3.4. Effect Size of Intervention Programs: Digital Devices, Exercise, and Music

This study conducted a random-effects meta-regression analysis to evaluate the effect size of the intervention programs’ characteristics on depression and cognition [30]. The results showed that exercise, medical facilities, number of sessions, and older age groups significantly affected depression. Exercise programs (*g* = −4.51, *p* < 0.05), medical facilities (*g* = −8.31, *p* < 0.001), and the number of sessions (*g* = −0.22, *p* < 0.05) had smaller effect sizes on depression. On the other hand, programs with older participants (the mean age of participants was 75 years or older) had a greater effect on depression (*g* = 8.07, *p* < 0.01). However, the effect size was not significantly affected by the digital devices and music intervention programs.

The results of the random-effect meta-regression analysis on cognition showed that digital devices and the number of sessions were significant intervention characteristics. Digital devices (*g* = −5.44, *p* < 0.05) had a smaller effect size on cognition. More sessions (*g* = 0.12, *p* < 0.05) increased the magnitude of the effect on cognition. Exercise and music did not significantly impact the effect size of the intervention programs.

## 4. Discussion

This study examined whether the effect size of intervention programs for the elderly with dementia that utilized digital devices, exercise, and music to alleviate depression and improve cognitive functions differed in comparison to those that did not. This study had an exploratory and practical character. It sought to facilitate the development and implementation of a program for the elderly with dementia that conducts exercise and music therapy (a drum instrument-playing game) using a digital device that combines virtual reality and haptic technology. The effect sizes of various programs for elderly Koreans on depression and cognition showed heterogeneity [14,18,23]. It was judged that the effect sizes were significant. The results of the meta-regression analysis revealed that intervention programs that utilized digital devices had a smaller effect size on the improvement of cognitive function than programs that did not use them, and their effect size on the alleviation of depression was not significantly different.

The results of the meta-regression analysis indicated that, even if digital device programs were found to significantly affect cognitive functions in a previous study, their effect sizes were relatively small compared to those of other intervention programs [13]. The effectiveness was evaluated in subjects whose cognitive function and depression were difficult to improve substantially, such as [13], using virtual reality on a small number of subjects (total eight subjects: four control subjects and four test subjects). It could be the reason why the effect size was relatively smaller than those of programs [19,32] that intervened with many participants in their 50s and 60s living in communities with normal cognition and depression. The authors of [13,16] also pointed out that dementia patients had various behavioral and psychological symptoms, and that it would be often difficult to improve their symptoms considerably by using non-pharmacological interventions. Although the results of this study indicated that the effect size of digital device intervention programs on cognitive functions was small, it did not imply that it had no value. A relatively small number of digital device programs were analyzed in this study––eight cognition-related programs and seven depression-related programs. This could indicate that digital device programs are still in a nascent developmental stage and possess tremendous potential for improvement [12].

The results of this study suggest that, as opposed to conducting repetitive activities that only emphasize the technology itself, it would be more effective to use digital devices to supplement other existing intervention programs or as an aid to a convergence program [13]. Ref. [16] also reported that, in terms of the dementia prevention programs, the integrated approach of the program curriculum, along with cognition and stimulation, had a significant impact on the effect size. It implied that it would be needed to develop programs with various and comprehensive activities by combining them with auxiliary digital equipment. Remarkably, although it had a small effect size on cognitive function, the effect size of digital device programs on depression was not significantly different from that of other programs [14]. This result can be interpreted positively, because the continued development of technology and the eventual reduction of the unit price may increase the efficiency of humans.

Exercise programs had a small effect size on depression, and their effect size on cognition was not significantly different. Since most existing exercise programs have been developed with the primary intention of maintaining participants’ physical function, they did not adequately consider the significance of ensuring that participants remained interested and happy [17,22]. Even though their effect size on depression was significant, it was interpreted to be smaller than those of other programs, such as those that incorporated play, music, gardening, art, or laughter [16,21]. This may be because exercise programs can be conducted without communication with other people in a narrow indoor space in a one-on-one setting or with a small number of people when exercise programs are carried out while interacting with other people and engaging in fun activities [17,33]. Therefore, there is a need to introduce a curriculum or equipment that can increase the effect size of exercise programs on depression while maintaining their effect size on cognition and physical activity [23]. In this respect, we propose a program that utilizes a digital device that incorporates strength exercises (through the performance of playing motions with drumsticks grafted with haptic technology) in virtual reality spaces where the elderly can feel comfortable or happy (e.g., outdoors, memorable places, or the sea). Furthermore, the device may enable them to sense the movement of small muscles.

This study’s meta-regression analysis revealed that the effect size of music programs on cognition and depression was not significantly different from that of other programs. The result did not mean that the music program was not effective, but that its impact was relatively small [16,23]. It is necessary to actively incorporate musical characteristics into other programs. It is desirable for music programs to pursue the effect of maintaining physical function [18]. However, playing real instruments involves many challenges, such as the difficulty in playing them, their high cost, and the weight of the instruments [21,22]. Dancing-oriented music programs also pose a high physical risk for the elderly who suffer from severe dementia or are admitted to facilities or hospitals [17,21]. Music must be incorporated in digital device and exercise programs for them to have a larger effect size on both cognition and depression [14]; music programs also need to pay attention to a new convergent approach [16]. Consequently, high efficiency and effectiveness can be achieved if the program is designed to involve the activity of playing music in a pleasant virtual reality space using equipment that offers exercise effects (e.g., drumsticks).

It was also found that the effectiveness of the intervention programs on cognition and depression varied based on their location (e.g., medical facility), number of sessions, and the age of the participants. The results were in alignment with those of previous meta-analysis studies [10,19,33], which reported that programs implemented in medical facilities had a small effect on depression, unlike on cognition. In contrast, the results of this study indicated that too many sessions decreased the program’s effect size on depression. In [33], the authors argued that the program should be implemented for more than 17 weeks, a long period, to have a large effect size on physical activity. It was a new finding that too many sessions were not effective for depression, unlike physical activity. The results imply that any new program should avoid conducting an excessive number of sessions. Since the effect size of the programs on the depression of the older-old (75 and above) was large, it might be suitable to provide programs more intensively to them. In summary, these results indicate that it is desirable to aim the program at the older-old, 20 sessions of which must be conducted in a medical facility, where any physical accidents due to dizziness and other causes can be prevented or immediately addressed.

The limitations of this study should be considered when applying its results. Directions for future studies are presented to address such limitations. Meta-analysis papers generally have standardized procedures, reporting order, and contents based on the PRISMA methodology. The methodology and results of this study are somewhat different from them. This study also tried to contain the contents as much as possible by referring to the topics and items of the PRISMA checklist. However, this study flexibly applied the format of the PRISMA because this study had two dependent variables and many programs to analyze (70 and 46), and complexity to deal with the results of meta-regression analysis. This study arbitrarily selected independent variables for exploratory purposes in the preliminary stage of developing exercise and music programs using digital devices. Although this study considered control variables based on the results of previous studies [18,19,22], it is possible that other characteristics that were not included in the meta-regression analysis model could change the estimates of the model. The difference between the effect sizes of some independent variables was not significant in the analysis results, possibly because the characteristics of intervention programs are not completely exclusive. Moreover, the effect size may vary if the analysis target is expanded to a wider variety of service agencies and community environments in countries with different socio-cultural backgrounds. Therefore, it is necessary to evaluate how the effect size changes when the program that we developed is implemented in the future and compare it to various existing programs.

## 5. Conclusions

We conducted a meta-regression analysis on intervention programs that compared the treatment group and the control group before and after their implementation to evaluate the effectiveness of interventions on the cognition and depression of the elderly in South Korea. The key results of this study revealed that the existing programs that incorporated digital devices had a small effect on cognitive function, whereas exercise programs had a small effect on depression. Another important result was that the effect sizes of digital device programs on depression, exercise programs on cognition, and music programs on depression and cognition were not different from those of other intervention programs. The results presented the hopeful possibility that a convergent program that combines music, exercise, and digital devices in a synergistic and unbiased manner could achieve a better effect size. The importance of this study is that its results can be utilized as a reference for facilitating academic and practical discussions for developing, implementing, monitoring, and evaluating improved programs. This study’s results can potentially facilitate the reduction of depression among the elderly and promote the maintenance and improvement of their cognitive functions.

## Figures and Tables

**Table 1:** Descriptive statistics about the characteristics of the examined intervention programs and their participants.

Variables	Depression		Cognition	
	*Mean* or *n*	% or *SD*	*Mean* or *n*	% or *SD*
Digital Device	46	100.0	70	100.0
Yes	7	15.2	8	11.4
No	39	84.8	62	88.6
Exercise	46	100.0	70	100.0
Yes	16	34.8	34	48.6
No	30	65.2	36	51.4
Music	46	100.0	70	100.0
Yes	15	32.6	23	32.9
No	31	67.4	47	67.1
Medical facility	46	100.0	70	100.0
Yes	23	50.0	37	52.9
No	23	50.0	33	47.1
Number of sessions	16.3	9.3	20.7	13.7
Gender	46	100.0	70	100.0
Women (50% or more)	36	78.3	58	82.9
Women (Less than 50%)	10	21.7	12	17.1
Age	46	100.0	70	100.0
75 years or older	31	67.4	43	61.4
Younger than 75 years	15	67.4	27	38.6

**Table 2:** Heterogeneity of the examined intervention programs.

Indicators	Depression		Cognition	
Hedges’s g (*z*) in the fixed effect	5.79 (46.77)	***	6.29 (40.74)	***
Hedges’s g (*z*) in the random effect	13.05 (17.87)	***	13.48 (15.22)	***
Cochran’s *Q* (*df*)	1005.77 (38)	***	1861.44 (62)	**
*I*^2^ value (Unit: *%*)	96.22		96.67	
*Tau* ^2^	44.11		39.66	

**Note.** ** *p* < 0.01. *** *p* < 0.001.

**Table 3:** Meta-regression analysis for evaluating the effect sizes of digital device programs, exercise programs, and music programs on depression and cognition.

Variables	Depression			Cognition		
	Coefficient		*SE*	Coefficient		*SE*
Digital device	−0.47		3.25	−5.43	*	2.51
Exercise	−4.51	*	2.69	−1.89		1.93
Music	1.03		2.66	0.95		1.87
Medical facility	−8.31	***	2.18	−1.21		1.72
Number of sessions	−0.22	*	0.12	0.12	*	0.07
Gender	0.11		0.07	3.03		2.26
Age	8.07	**	2.65	1.52		1.81
Intercept	10.27	*	5.81	9.31	***	2.62
*Tau* ^2^	44.11			39.66		
*I* ^2^	96.22			96.67		
Q (*df*)	1005.77 (38) ***			1861.44 (62) ***		
*n*	46			70		

**Note.** * *p* < 0.05. ** *p* < 0.01. *** *p* < 0.001. One-Tailed tests.
